# Interactions of Technology and Obsessive-Compulsive Disorder Symptomatology in Adults: Qualitative Interview Study

**DOI:** 10.2196/85033

**Published:** 2026-02-05

**Authors:** Lucas Occhino-Moede, Kaitlyn Sulivan-Pascual, Kendall Phelan, Harrison Wang, Daniel Mokhtar, Elisa Liu, Erica Schug, Megan Mirkis, Thomas Baek, Tamerlane Visher, Ujjwal Pasupulety, Adam Charles Frank

**Affiliations:** 1 Department of Psychiatry and Behavioral Sciences, Keck School of Medicine, University of Southern California, 2250 Alcazar St., Suite 2200, Los Angeles, CA, 90033, United States, 1 323-442-6000

**Keywords:** obsessive-compulsive disorder, OCD, technology, wearables, qualitative, patient perspectives

## Abstract

**Background:**

Obsessive-compulsive disorder (OCD) affects 1%‐3% of the population and is marked by intrusive obsessions and compulsive behaviors that impair daily functioning. As digital technologies have become ubiquitous, their features may interact with OCD symptom dimensions in ways that both exacerbate and alleviate symptoms. While case reports and clinical anecdotes suggest such interactions, systematic investigation of patients’ lived experiences with technology remains limited.

**Objective:**

This study aimed to explore how individuals with OCD perceive and navigate their interactions with modern technologies, and to identify how specific features of technology may contribute to, reinforce, or relieve obsessive-compulsive symptom cycles.

**Methods:**

We conducted semistructured interviews (n=24) with adults self-reporting a diagnosis of OCD, recruited through online OCD communities and advocacy networks. Interviews were conducted via the HIPAA (Health Insurance Portability and Accountability Act)-compliant platform Zoom (Zoom Communications) between May and December 2024 (median duration 51, IQR 6.5 minutes). Transcripts were coded in Dedoose (version 9.2.22; SocioCultural Research Consultants) using a constructivist grounded theory approach. Coding proceeded iteratively through open and focused coding, with theoretical saturation reached after 15 interviews. Constant comparison and analytic memoing guided the development of a conceptual framework linking technology features to OCD symptom dimensions.

**Results:**

Participants (median age 26, IQR 12.8, range 20‐64 years; 67%, 16/24 women, 29%, 7/24 men, and 4%, 1/24 nonbinary) described technology as both a trigger for and a coping tool against OCD symptoms. Analysis produced four central technology-related categories: (1) information-provision platforms (eg, social media, search engines, large language models, etc) that triggered disturbing-thought obsessions and enabled compulsive checking and reassurance-seeking; (2) gamification and quantification features (eg, streaks, progress bars, and tracking metrics) that reinforced “not-just-right” and symmetry-based compulsions; (3) notifications that provoked urges to clear, check, and maintain control, spanning both disturbing-thought and symmetry domains; and (4) user interfaces whose complexity and customizability elicited compulsive ordering, avoidance behaviors, and digital overwhelm.

**Conclusions:**

This study characterizes how interactions between OCD and digital technologies manifest across established symptom domains, most notably disturbing-thought and “not-just-right” categories. Participants overwhelmingly experienced compulsive checking, reassurance-seeking, and ordering behaviors reinforced by features such as information-provision, gamification, notifications, and user interfaces. These findings highlight the clinical relevance of technology-related compulsions and suggest value in their systematic assessment, incorporation into psychoeducation, and consideration in digital design.

## Introduction

Obsessive-compulsive disorder (OCD) occurs in 1%‐3% of the population [1] and is most commonly characterized by the presence of obsessions and compulsions. Obsessions are intrusive, unwanted thoughts, images, or impulses that are typically experienced as ego-dystonic and distressing. Compulsions are repetitive actions or mental rituals in which an individual engages to relieve anxiety related to obsessions. Obsessions and compulsions, in the context of OCD, are time consuming and often decrease functioning in social, occupational, and other aspects of life.

OCD is a heterogeneous disorder with variety in the types of obsessions and compulsions experienced, severity of symptoms, and presence of comorbidities. While no two individuals are likely to experience the same pattern of symptoms, factor analytic approaches have been used to parse OCD heterogeneity. These studies have consistently identified the following four broad dimensions: (1) forbidden or taboo thoughts, (2) symmetry and ordering, (3) contamination and cleaning, and (4) hoarding [2,3]. More recent research has used expanded symptom checklists and refined analytic techniques to explore the relationships between these dimensions. The higher-level groupings of incompleteness and disturbing thoughts (distressing images that typically follow themes like contamination, harmful acts, or religion) strongly associate with the other dimensions and thereby are postulated to represent core phenotypes within OCD. Subdimensions within these two categories were also delineated and noted to be structured in a hierarchy. The first order domain of incompleteness was divided into accuracy, mental and perceptual, and “not-just-right” (NJR) behaviors. NJR experiences are typically an internal sense of disturbance related to qualities such as order, placement, or frequency and drive individuals to engage in actions like tapping, counting, or reviewing. Meanwhile, disturbing thoughts encompassed symptoms relating to harm and checking and forbidden thoughts [4].

Excessive or inappropriate use of technology is associated with mental health issues. Children and adolescents are particularly susceptible; higher amounts of screen time and social media use have been linked to a range of mental health symptoms due to disturbances in regular sleep-wake cycles [5]. Technology can also facilitate maladaptive behaviors such as compulsive internet use and gambling disorder [6]. Patients with psychotic disorders have expressed that online platforms can exacerbate their paranoia and delusions through misinformation or targeted harassment [7]. Within OCD, the increasing digitization of society over the past several decades has provided new mediums for the manifestation of symptoms [8]. Recent case reports describe patients with OCD whose symptoms have been exacerbated by technology use. One individual described obsessions about inappropriately reacting to social media posts and would consequently spend hours per day scrolling through her feed to confirm that she had not mistakenly done so. Another patient worried that she had posted shameful content online and would screen record her internet use to later verify appropriate use [9].

However, advances in digital health have also introduced a wide range of technologies for the assessment and management of OCD. Passive monitoring tools, such as actigraphy and wearable devices, have been used to unobtrusively track sleep and activity patterns, often aligning closely with patient reports [10]. Active approaches, including ecological momentary assessment, allow for real-time symptom capture through smartphone prompts, offering insights into daily fluctuations that may be missed by retrospective recall [11]. Beyond monitoring, emerging interventions incorporate smartphone-based apps, cognitive training tools, and even multimodal systems that combine neurophysiological measures with behavioral data [12].

There exists a need to further understand patients’ perspectives toward technology and how its design or features might interact with their OCD. It remains unclear what forms of technology are more likely to trigger obsessions or compulsions, how these symptoms manifest, and whether patients find such interactions distressing or relieving. This qualitative study aims to address these questions by examining the mechanisms through which technology may contribute to or reinforce obsessive-compulsive behaviors.

## Methods

### Study Design: Constructivist Grounded Theory

We conducted a phenomenological study aimed at understanding the ways in which individuals with lived experience of OCD uniquely interface and interact with technology. Methodology was guided by constructivist grounded theory, which frames analysis as dialogic and iterative, constructing an empirically grounded model of the phenomenon under study [13]. We used the COREQ (Consolidated Criteria for Reporting Qualitative Research) framework, a validated checklist for qualitative reporting, to guide and document important aspects of the research team, methodology, findings, and analysis [14]. The study was approved by the University of Southern California Institutional Review Board (UP-23-01094).

### Recruitment and Sample

Participants were recruited through a convenience sampling method between May and December 2024 through online posts in OCD-related communities, including the International OCD Foundation (IOCDF) website, OCD SoCal (a local affiliate of the IOCDF), and existing OCD-focused research participant registries maintained by the laboratory. These online spaces included both moderated forums and institutional web pages aimed at providing information, treatment resources, or research participation opportunities for those with OCD. Recruitment materials included a standardized study announcement containing the study overview, eligibility requirements, and compensation details. Potential participants were routed to contact the study team via email for screening through a REDCap (Research Electronic Data Capture; Vanderbilt University) survey that gathered demographic and self-report data relevant to the inclusion criteria. Study personnel reviewed survey responses in order of submission and contacted participants who met study criteria to schedule an initial virtual meeting to confirm survey responses and obtain informed consent. Following completion of the study, participants were compensated with a US $50 gift card for their time and effort. Inclusion criteria included self-reported age of 18 years and older and a diagnosis of OCD. A total of 27 participants completed the consent process and were interviewed. All interviews were reviewed by study personnel trained in the phenomenology of OCD for content consistent with knowledge of and experience with OCD. A total of 3 interviews were excluded due to credible concerns regarding eligibility (eg, no clear indication of an OCD diagnosis and suspected repeat participation by a single individual). To mitigate this risk, we subsequently implemented procedures requiring camera-on interviews and a brief discussion of participants’ OCD symptoms with the interviewer prior to proceeding. No participants withdrew or declined participation after enrollment. A total of 7 participants had previous contact with laboratory personnel through other unrelated research studies.

### Research Team

Data collection and analysis were conducted by a multidisciplinary team, including the principal investigator (ACF; MD, PhD, psychiatrist) and graduate and undergraduate research assistants. Multimedia Appendix 1 provides an overview of team demographics, training, and specific roles. Interviewers disclosed their role in the laboratory to participants at the beginning of each interview. Team members brought varied clinical, academic, and personal perspectives related to OCD and technology and had differing levels of qualitative research experience.

To ensure consistency and competency, all interviewers completed a minimum of 6 weeks of qualitative research training. Standardization efforts included review sessions of the interview guide, observation of senior interviewers, and postinterview debriefings.

### Data Collection and Setting

Interviews were conducted via the HIPAA (Health Insurance Portability and Accountability Act)-compliant Zoom software (Zoom Communications) between May 16, 2024, and December 20, 2024. A primary facilitator led each interview, with additional team members present to assist with technical support or occasional follow-up questions. Only study participants and research team members were present during interviews. Interviews ranged from 35 to 65 minutes, with a median duration of 51 minutes.

All interviews were audio- and video-recorded, and transcripts were generated using Zoom’s auto-transcription feature. Research assistants verified transcript accuracy and performed deidentification. All audio and video recordings were permanently deleted after transcript verification. No repeat interviews or member checking of transcripts were conducted.

### Interview Guide

The semistructured interview guide was developed to explore participants’ lived experiences with technologies used for health or health care, particularly in relation to OCD. The guide was informed by phenomenological and user-centered perspectives, emphasizing discovery, motivation, use patterns, benefits, concerns, and perceived interactions with OCD symptoms. While not based on a single theoretical model, the structure reflects elements common to sociotechnical and technology adoption frameworks, with a focus on how individuals make sense of and navigate these tools in daily life [15,16]. Interview questions were piloted within the research team and refined through early interviews as part of our grounded theory approach. Revisions were made iteratively as team discussions and early analysis revealed new areas of interest. A sample of the interview guide is provided in Multimedia Appendix 2.

### Data Analysis: Coding

The first 5 transcripts were independently open-coded by pairs of researchers, where open-coding refers to the initial process of breaking data into discrete parts and assigning conceptual labels to segments of interest. Each pair met to discuss initial findings and reach consensus on a shared set of open codes for each transcript. These were then reviewed through group meetings to identify patterns and groupings, generating a set of focused codes informed by emerging theoretical insights that constructed the initial codebook. The coding structure is outlined in Multimedia Appendix 3. Transcripts were uploaded to Dedoose (version 9.2.22; Sociocultural Research Consultants), and the codebook was implemented in the software. The codebook was then applied to each transcript by a pair of researchers, and consensus meetings between coders followed to ensure agreement on code applications.

We operationalized theoretical saturation in our dataset by the inclusion of a “new finding” code in the codebook to capture data relevant to the research question but not represented by existing codes. After all transcripts were coded, the “new finding” excerpts were reviewed collectively to assess whether additional conceptual categories had emerged. New categories were tracked and subsequent emergence of related insights was collapsed, such that “new finding” applications represented only unique and uncaptured theoretical insights [17,18]. We considered interview number 15 the point of theoretical saturation as no subsequent “new finding” applications identified categories or relationships that would meaningfully alter the emerging theory (Multimedia Appendix 4). These discussions led to final adjustments to the codebook. All transcripts were then recoded retrospectively using the updated coding structure.

### Data Analysis: Theory Generation

After applying the final codebook, the team engaged in an inductive analytic process to develop a theoretical framework describing a salient emerging topic from the data: understanding interactions between technology and OCD symptoms. Using constant comparison across transcripts and memos, we identified key conceptual categories, examined their interrelationships, and developed an articulation of the processes shaping participants’ experiences.

Analysis began with targeted review of excerpts coded with conceptually related codes (“Exacerbating and enabling OCD symptoms with technology” and “Alleviating mental health and OCD symptoms with technology”). These were used to write analytical memos, which guided weekly team discussions and supported theoretical elaboration. Through this iterative process, we developed a conceptual framework that articulates relationships between specific types of technology, the symptom domains they interact with, and distinct stages of the OCD symptom cycle.

### Ethical Considerations

The study was approved by the University of Southern California Institutional Review Board (UP-23‐01094). All study procedures involving human participants adhered to the ethical standards of the institutional review board and the 1964 Declaration of Helsinki and its subsequent amendments. All subjects received written information regarding study aims, procedures, potential risks, and anticipated benefits, which were reviewed in discussion with study personnel during the onboarding process. All participants provided informed consent before study enrollment. Participation was voluntary, and participants could withdraw at any time without penalty. Participant privacy and confidentiality were strictly protected, and all data were deidentified before analysis. Participants were compensated with a US $50 Tango gift card upon study completion.

## Results

### Participants

A description of the participants (n=24) is provided in Table 1. No participants refused to participate in the study or withdrew their participation.

**Table 1.** Participant demographics (N=24).

Characteristics	Results
Age (years), median (range)	26.2 (20.1‐63.6)
Gender, n (%)	
Men	7 (29)
Women	16 (67)
Nonbinary	1 (4)
State, n (%)	
California	14 (58)
Pennsylvania	3 (13)
Illinois	2 (8)
Ohio	2 (8)
Florida	1 (4)
Maryland	1 (4)
Virginia	1 (4)

A coding tree with 5 parent codes and 7 child codes was developed during analysis. Among these, the parent codes Exacerbating and enabling OCD symptoms with technology and Alleviating OCD symptoms with technology were most central to addressing the research question and guided the organization of findings. From this coding structure, we identified four pertinent qualities of technology in relation to participants’ symptomatic experiences: (1) information provision, (2) gamification and quantification, (3) notifications, and (4) user interfaces. Information-provision technologies had strong interactions with OCD symptoms consistent with the disturbing-thought domain, whereas the 3 other technology characteristics had interactions with both disturbing-thought and symmetry domains. Notably, the application of OCD symptom domains (eg, symmetry and disturbing–thought-based) was not deductively imposed during the interview process or initial codebook development. Rather, these domains were inductively derived from participants’ narratives and symptom descriptions. While the interactions between technological characteristics and symptom manifestations emerged organically from the data, the terminology used to describe these domains is drawn from the existing OCD literature to ensure consistency and scientific clarity in reporting [2,3].

### Result 1: Information-Provision Technologies and the Disturbing-Thought Domain of OCD

Across interviews, participants described information-providing technologies as implicated in the emergence and reinforcement of symptoms within the disturbing thought-based domain of OCD. These technologies, which range from social media and search engines to health portals and messaging apps, triggered intrusive, ego-dystonic thoughts, while also enabling compulsive behaviors such as checking, reassurance-seeking, and mental review.

Many participants emphasized the role of unsolicited and algorithm-driven content in triggering disturbing-thought symptoms. These triggers were often delivered unexpectedly via video thumbnails, pop-up ads, or headlines, bypassing user intention and presenting emotionally charged or ambiguous material. This dynamic was especially distressing for individuals with harm, health, or moral scrupulosity themes:

It just pops up on your page. It can be very triggering….I started getting videos of this one specific food that I really like… This patient that was eating this food a lot developed fatty liver disease and my OCD brain was like, ‘oh no’… I’ve been spending hours every day since seeing that, you know, researching correct dietary choices, researching health.

[P3]

The above quote also highlights the role of information-providing technology in inducing compulsive checking or reviewing to gain certainty or reassurance in response to obsessional distress. In our data, obsessional distress was related to intrusive fears of past wrongdoing, undiagnosed illness, or social harm. Commonly implicated platforms used for checking or reviewing included search engines, social media timelines, court databases, and health information portals:

*I will get on my phone…look on social media for like dates and look up people and you know, think that I hurt this person or did I do this? Did I do that?…I’ll look my name up on the sex offender registry and I’ll be like, oh no, you know, am I on here?…all that information being out there just makes me want to search and search more.*

[P2]

Technologies that allowed participants to upload information for others to interact with produced similar patterns in both triggering disturbing-thought obsessions and enabling compulsions. Written communication platforms such as email and messaging apps were particularly salient for participants with social or moral scrupulosity, a subtype of disturbing-thought OCD. Participants reported that the informational permanence of these technologies often led to obsessive worries about their perception by others, including harm, misunderstanding, or being morally inappropriate. Participants then described compulsively reviewing sent messages, analyzing others’ tone, and fearing reputational or ethical harm:

*Yes. I had, I guess I had some OCD or some anxiety anyway about the Teams chat at work that we use for workplace communication…I would go back and check to see…could this be taken [as] inappropriate by this person? And I'll, you know, check, check a few times*.

[P1]

Participants who used information-providing technology compulsively often acknowledged that the boundaries between reassurance-seeking, educational inquiry, and compulsive overuse are often opaque:

*I just compulsively look up health care information…I think initially like all compulsions, when they’re carried out, it provides some reassurance, although that’s not always healthy*.

[P1]

In some cases, participants reported that an obsession-compulsion cycle would subside once they encountered information that felt sufficiently reassuring. However, the threshold for what constituted a “sufficient” or trustworthy source varied across individuals. Both participants P8 and P9 described using search engines to manage health-related obsessions but differed in their sense of reassurance from the information they found:

*So, I guess just try to, like, put it out of my mind until I can actually talk with someone from the office. So just, like, distract myself, or I’ll find something on the internet that will maybe explain it, if I feel like it, is the answer. But usually I’m never like. Usually I’m never satisfied, because, like different websites can say different things. So I mean, that can sometimes work like, if I feel like, okay, I have a reliable website, and you know it says this. Then I’ll feel like I found, you know I have the answer or the explanation to it*.

[P8]

*So like, some websites are more trusted than others…But again…I have trust issues deep down. And so the information I’m getting, I don’t feel confident in even though I go to town. As soon as I see it, I kind of feel better because, you know, it answers my question. Like I just got a question answered, which is an anxiety, like to not know…Then it’s like, I need to keep going and look, look, look, look. And I go to multiple different lengths. So I guess there’s really not a point where I feel confident in it. And because the point is that my brain goes into more and more and more possibilities…And then I end up just having a bunch of different things that could be wrong with me. Um, and then I message my doctor and…that’s pretty much how that goes*.

[P9]

The duality of relief and exacerbation that informational technologies evoke was complex and individualized. The type of clarifying or validating information was also varied and included peer connection, digital monitoring, and access to authoritative sources. Another nuanced experience of participants regarded their deliberate limiting or avoidance of technology platforms perceived as triggering. For some, this involved active efforts to reduce exposure to social media or other content-driven apps:

Yeah, social media, like, I have Instagram, kind of only of the social medias, and it’s a constant fight. I try to use it as little as I can, down to like, I delete the app every few days…That interacts for sure with my OCD.

[P7]

However, participants also reflected on the complexity of avoidance itself. While reducing exposure to distressing content was sometimes framed as protective, others were careful to distinguish between harmful avoidance (which can reinforce OCD) and what they considered healthy boundary-setting:

I guess if we want to talk about media… I try to avoid the amount. And I shouldn’t really say ‘avoid,’ I guess, ’cause that makes it sound like counterproductive avoidance, but more like a self-care kind of avoidance, if you will…I will kind of doom spiral and ruminate and obsess about things like that if I focus on it too much…I just wanted to clarify, not be counterproductive. As we all know, like, avoiding things to heal your OCD doesn’t work. That just makes it worse in the long run. So that’s why I don’t mean like hiding away from it, I mean just having my own healthy boundaries with things.

[P5]

Across both positive and cautionary accounts, participants consistently emphasized how the structure and function of information-providing technologies, whether through unsolicited content or user-driven inquiry, directly shaped the phenomenology of their disturbing-thought–based OCD symptoms.

### Result 2: Gamified and Quantified Technologies in Symmetry- and Disturbing-Thought–Based OCD

Several participants described how technologies that quantify behavior or incorporate gamified features—such as streaks, scores, and progress tracking—interacted with their OCD symptoms. These interactions most commonly aligned with symmetry- or NJR-based obsessions. In these cases, discomfort was often triggered by an unbalanced number, an incomplete goal, or an interrupted streak and was accompanied by a vague but compelling internal sense that something was unresolved. Several participants expressed this interaction with health and fitness tracking apps:

*Sometimes, you are too addicted to getting the perfect [step count] scores…I need to achieve this target, although you might have been drained out and tired throughout the day. But you want to. You want that number to be complete in order to just, like, tick off. It feels like ticking off one of the checkboxes.*

[P13]

Participants frequently acknowledged that the distress did not always stem from a clearly articulated fear or consequence. Instead, their behaviors were driven by an ambiguous internal pressure, with goals or numbers taking on a rigid symbolic weight:

I would say I’m kind of obsessed with trying to have at least the same or more number of steps every day… I didn’t think about that, but that’s absolutely true… So I have like, let’s say 10,000 or 11,000 steps a day and when I see that it’s less, I’m like, oh, no, you know, I need to go for a walk and just go get some groceries just to make it even neater…I check it very often… so that gives me some anxiety probably you know internally…I can’t figure out, I mean, the thoughts behind that, like the obsession…

[P15]

The automatic provision of a step count number to the user both initiates distress and enables individuals to perform checking compulsions. This highlights the ambiguity often described by participants in relation to NJR feelings with a fixation on numbers and achievements. One participant powerfully captured how app-based gamification and quantification intersected with OCD perfectionism in a way that borders on overwhelming:

Anytime when there’s a streak, like you’ve meditated 9 times in a row, or like the way the apps are kind of gamified. The worst for this is Duolingo…just to give an example where it’s like, you have all the cauldrons of stars and bonuses and streaks, and to me, that adds a layer of gamification and stress and numbers and scoring. And related to my OCD perfectionism, that’s not as constructive to me…I would say it interacts…when it seems to be a thing I can pass and fail at. Particularly because there are numbers involved and or a kind of like, gamified system…when it feels like that kind of contest…It makes it more complicated.

[P7]

While symmetry-related themes were most common in response to these technologies, some participants described gamified or quantified features as triggering distress related to disturbing-thought–based obsessions. In these cases, the compulsion was not driven by a need for balance or completion, but rather by intrusive fears about consequences if the behavior was not completed:

I used the Headspace app to just…calm the thoughts…, but it ironically turned into kind of an OCD pattern where I felt like I couldn’t not do the Headspace thing…I remember I had like a 130-day streak, which is great. But I realized that there were some days where I didn’t want to do it…[My OCD] kind of left me in a pattern where it was like, you need to do it, or you’re gonna fall back, or you’re gonna retreat to your old self…The first therapist I met with was the one who kind of helped me be like, it’s okay if you miss a day or two—like, the world’s not gonna end.

[P14]

This development of intrusive fears and compulsive use of a mental health app underscores both the seemingly innocuous sources of symptom onset and the dual potential of mental health technologies to be either helpful or harmful, depending on how they interact with a user’s symptom profile.

Some participants note that there are qualities of quantification technology that exacerbate obsessive-compulsive symptomatology in ways that nondigital alternatives might not:

MyFitnessPal was the one where I realized, okay, I need to stop using this…everything I ate, I had to put in. I spent a bunch of time making sure I found as accurate as possible of a submission for what I ate that day…because if you’re just journaling, you’re not going to see your progress towards this [amount of calories], like if you’ve gone over by this amount…So it was just very automatic and specific. And I think that’s kind of what led to it. Versus if I was journaling, I don’t think I would have been as likely to be tracking it so tight.

[P11]

I can find myself like, really obsessed with my reading speed and the percentage that I finish a book. So, you can track both your reading speed and the percentage that you finish a book on Kindle versus, you know, paper, and I can find myself feeling stressed or, like, feeling unable to do anything else unless I complete my reading goal. Like, I make my reading goal to reach like 5% of book, for example. And I feel like I cannot stop reading until that’s done and that’s only on kindle. It’s such a quantified thing.

[P4]

Together, these accounts illustrate how features like quantification, gamification, and automated feedback can interact with both symmetry-based and disturbing-thought-based OCD symptoms, transforming everyday technologies into sources of obsessional triggers and compulsive pressure.

### Result 3: Notifications and the Urge to Clear, Know, and Control

Participants frequently described notifications as a trigger for compulsive interactions that spanned both NJR- and disturbing-thought–based OCD experiences. Participants described two distinct ways notifications interacted with OCD symptoms. For some, notifications triggered a need for visual or numerical resolution such as clearing unread counts to zero or removing banners that disrupted the screen, which aligned with NJR experiences. For others, notifications provoked disturbing-thought–related fears about neglecting responsibilities or missing important information, reinforcing compulsive checking behaviors.

For participants whose distress aligned with symmetry-based experiences, notifications disrupted a visual or emotional sense of order:

I also have to make sure all the notification numbers on the apps are cleared every time I check it…my wallpaper is a graduation photo with my parents. If a notification pops up and covers my mom, I have to remove it to make sure the important parts of the photo are not covered.

[P6]

I get really overwhelmed by my computer and my phone and in relation to my work. It’s a space of like, Slack messages that I’m obsessive about, and clearing my inbox to 0 and, you know, checking my email again and again… It becomes like interface [number] 13, that I need to have like cleared and sort of dealt with.

[P7]

For this participant, the presence of notifications created a sense of visual clutter or incompleteness that became intolerable, prompting frequent checking and clearing rituals.

Other participants described notification-related distress that aligned more closely with disturbing-thought–based OCD, particularly around fears of neglecting responsibilities or missing something socially or professionally important. The spontaneity and unpredictability of notifications compounded the sense of being overwhelmed.

I just feel a bit burdened about it. Oh, I have a lot of notifications to clear, I need to catch up on, just so that I’m informed. Oh, okay, all of these are there, and I’ve read through them. So at least I have the knowledge of what was in my notifications.

[P10]

This compulsion of checking and clearing reflects a drive to restore a sense of control by accounting for and resolving the uncertainty that notifications generate.

### Result 4: User Interfaces and Experiences of Ordering and Overwhelm

Participants described how the interfaces of personal devices frequently triggered obsessive-compulsive symptoms. Features typically celebrated for enhancing usability, such as customization, integration, and expansive functionality, were often experienced as overwhelming or destabilizing. For individuals with OCD, these features presented countless opportunities to engage in compulsions related to control, ordering, and avoidance, particularly when the interface felt overly stimulating or difficult to contain. These experiences aligned with both symmetry and disturbing-thought–based OCD symptom domains.

For example, some participants described compulsive ordering rituals related to app layout and usage patterns:

So, in my OCD, there’s a certain way I do things, like there’s a certain way I wash my hands. So similarly for social media, I have a certain order in which I use the apps and a certain order and amount of time. On every app there is a routine for it, like, for example, Whenever I wake up, I'll first open Instagram, then LinkedIn, then Handshake, then maybe Snapchat. So, there is a particular order.

[P10]

While certain participants engaged compulsively with interfaces in ways that clearly aligned with symmetry-based symptoms, such as imposing order, others described distress in response to the layered, limitless nature of digital environments. These experiences were characterized by feelings of cognitive overload or a sense of losing control. However, the underlying sources of this distress were more difficult to categorize. For some participants, it appeared to reflect a symmetry-based desire for structure, simplicity, or containment. A participant described this experience while engaging with cloud-based storage systems:

If it’s my file, it’s just abstract. It’s weirdly hard for me how everything is virtual, just like not having that order…I work with one organization where it’s all through Google Drive. There are hundreds and hundreds of sub folders…Getting lost in these online labyrinths somehow overwhelms my OCD. I struggle when I can’t, like, control. And maybe this speaks to apps in terms of simplicity of interface for me. I think that would be more soothing to my OCD, because it wouldn't have that effect like, “this is a thing with like 10 subfolders, and so many different places to explore”…Yeah, if there’s too much to it, it can be overwhelming.

[P7]

This sense of digital overload prompted some participants to deliberately restrict the functionality of their devices or to seek out simplified alternatives. The goal was not merely to reduce screen time, but to avoid the sense of overstimulation caused especially by vast integration of technology. A participant reflected on the difference between two wearable devices, seemingly driven by a disturbing-thought fear such as missing important information or falling behind:

There’s so many different ways to reach me. So that is something that I get very, internally stressed and overwhelmed with…That’s one of my main symptoms, is that feeling of always having to catch up and keep up with all the side conversations [on my phone]…The Apple watch reflected my computer, my phone, everything, on my wrist, which was just too much for me. I find the Fitbit to be less invasive because I haven’t fully integrated my whole phone into it. I really enjoy the Fitbit for the limited functions that I’ve got on it right now. I know that it has more capabilities, but I like that I haven’t turned those on.

[P12]

Across interviews, participants described how the boundless, integrated nature of digital interfaces transformed their devices into overwhelming, even invasive, objects. Abundance itself became a source of distress as the increasing complexity and functionality of user interfaces created countless opportunities for engagement that could both facilitate compulsions and trigger anxiety. For some, these technologies reinforced ordering or avoidance rituals; for others, they produced a chaotic sense of overstimulation and loss of control. Several individuals noted limiting or simplifying device functions as a self-protective strategy to reduce compulsive urges and restore a sense of control.

## Discussion

### Principal Findings

Our findings show that everyday technologies shape the expression, maintenance, and phenomenology of OCD in ways that are both domain-specific and cross-cutting. Information-providing technologies often amplified disturbing-thought symptoms by delivering unexpected or ambiguous content and by enabling compulsive reassurance-seeking, checking, and mental reviewing. Participants described a fluid boundary between appropriate information gathering and compulsive overuse, noting that algorithmic content, the permanence of written communication, and the abundance of online information all contributed to escalating cycles of uncertainty and distress.

Gamified and quantified technologies interacted with symmetry- and NJR-based symptoms by attaching symbolic weight to numbers, streaks, and completion metrics. These features provoked both an internal drive for balance or perfection and, in some cases, intrusive fears about the consequences of breaking a streak. Participants described how automatic feedback loops transformed neutral metrics into triggers for compulsive behavior.

Across symptom domains, notifications acted as potent cues for urges to clear, control, and know. Some participants experienced notifications as visual disorders that required resolution, while others felt compelled to check notifications out of fear they might overlook or miss essential information. These experiences were reflective of both symmetry-based obsessions and disturbing-thought-based obsessions.

Finally, the structure of the user interface itself emerged as a significant source of OCD-related distress. Highly integrated or complex digital environments elicited compulsive ordering, avoidance, or feelings of cognitive overload. Many participants sought simplified devices, simplified digital environments, or limited device functionality to regain a sense of control and reduce compulsions.

Together, these results illustrate how contemporary technologies deliver content and structure interactions in ways that directly shape OCD phenomenology. The design of digital environments, including informational density, automated feedback systems, and levels of integration across platforms, creates conditions in which obsessions can be triggered and compulsions reinforced. These findings point to technology as an active context in which OCD symptoms unfold, rather than a neutral backdrop. This perspective helps clarify why existing research has only begun to capture the breadth and nuance of these experiences, and it provides a foundation for situating our results within the emerging literature.

### Previous Research

There is a relative dearth of literature exploring stakeholder perspectives on how OCD interacts with technology, both as a mental health disorder and a lived experience. Previous work has highlighted how symptoms could manifest in the digital realm, including intrusive worries about posting offensive content to the Internet or social media, with associated compulsive checking, screen capture, and reassurance seeking [9]. Expert opinion now suggests assessing for “digital obsessions and compulsions,” when evaluating patients with OCD [8]. A number of studies that used technology in the assessment and treatment of OCD also asked participants about their experiences using this technology; however, this information was not collected systematically and remained broadly focused on acceptability and tolerability [19-21]. Finally, one study explored experiences with online peer-support communities in individuals with OCD; thematic analysis revealed factors such as social comparison and misinformation as contributing to negative experiences [22].

This study sought to explore how individuals with OCD perceive and navigate their interactions with modern technology. Notably, neither our interview guide nor codebook was structured to map onto formal OCD dimensions such as those delineated by the Yale-Brown Obsessive Compulsive Scale Symptom Checklist [23]. Yet, clear and consistent patterns emerged that aligned with established symptom clusters, most notably the disturbing-thought (eg, harm, sexual, religious, and moral scrupulosity) and NJR domains. Participants described experiences that mirrored clinical subtypes, underscoring the validity of these dimensions and the extent to which they shape real-world behavior, including digital behavior.

Interestingly, symptoms in the domains of cleanliness, contamination, and excessive or ritualized washing or grooming were less prominent in our data. This absence may reflect that contamination concerns often center on physical contact with contaminants and health-related concerns generally have a focus on the physical body. However, mental contamination is a recognized phenomenon in OCD and shares features with contact contamination symptoms and symmetry and incompleteness symptoms [24]. While this was not present in our data, interactions between technology and mental contamination should be considered for further exploration. Finally, our findings complicate assumptions that certain domains are purely physical. For example, other studies have found that digital clutter can provoke similar emotional distress and difficulty discarding as does physical hoarding [25].

Below, we elaborate on the implications of these findings for 4 key stakeholder groups: patients, clinicians, technology developers, and researchers.

### Implications for Patients

Technology can serve as an important source of community, a method for accessing educational and treatment resources, and an approach to monitoring symptoms for individuals with OCD. However, these same tools can exacerbate symptoms and complicate treatment. Patients may benefit from intentionally monitoring how digital tools affect their emotional state and symptom patterns over time. We recommend that individuals reflect on the distinction between purposeful use of technology and patterns of behavior that drift into compulsive checking, reassurance seeking, or overmonitoring.

In practical terms, this may involve setting time- or context-based limits, pausing engagement with certain platforms during periods of heightened vulnerability, or discussing emerging online behaviors with a therapist to identify early signs of compulsive use. Patients should also be mindful that customization features, achievement systems, and constant access to information can shift from motivating to destabilizing without clear warning. We suggest open and ongoing discussions between patients and their treating clinicians regarding technology in daily life and OCD treatment.

We suggest framing technology not as inherently helpful or harmful, but as a continuum of use that can shift depending on symptom severity, emotional needs, and the design of specific platforms. Approaching digital engagement with this flexible, self-reflective mindset may help patients cultivate healthier, more sustainable relationships with the technologies that shape their daily lives.

### Implications for Clinicians

For clinicians, assessment of OCD symptomatology is important, and we concur with previous studies that recommend explicit evaluation for digital behaviors [8]. This can be achieved by adding targeted questions (eg, “Do you feel compelled to check, post, or search online to relieve distress, even when it interferes with your life?”) or by supplementing existing measures with a brief checklist addressing technology-related compulsions, such as repetitive searching, reassurance-seeking, or monitoring notifications. Because current instruments do not systematically probe for these behaviors, clinicians should remain attentive to technology use that functions as a compulsion. It also remains unclear what proportion of OCD symptoms can be attributed uniquely to digital compulsions, and future studies should quantify this domain to inform updates of assessment tools.

With regard to treatment, clinicians should incorporate psychoeducation about how app design, social media features, or online communities can inadvertently reinforce obsessive-compulsive cycles. We suggest clinicians provide concrete examples to patients: streaks and push notifications can induce checking behaviors, social media can reinforce negative stereotypes and foster misinformation, and user interfaces can lead to a feeling of digital overwhelm. These examples can help patients recognize their own experiences and better understand these processes as environmental triggers rather than personal failings. Clinicians might also help patients identify strategies for more adaptive engagement, such as disabling notifications, setting time limits, or designating “offline” periods.

Cognitive-behavioral strategies, particularly exposure and response prevention, can be readily adapted to digital contexts. For instance, therapists might collaborate with patients to create graded exposure hierarchies that include delaying responses to notifications, resisting the urge to refresh a feed, or intentionally interrupting a streak or posting schedule. These exercises can be integrated into therapy sessions or assigned as structured homework.

Finally, clinicians should maintain flexibility as technology evolves. Integrating a brief review of digital behaviors into routine follow-up visits can help identify emerging risks, prevent relapse, and reinforce adaptive digital habits. Tracking these observations in deidentified case notes may also contribute valuable data for refining diagnostic instruments and treatment protocols.

### Implications for Technology Developers

Our findings raise important considerations for those who design and develop digital platforms. Many features intended to enhance engagement, such as algorithmic personalization, goal-setting interfaces, streak counters, and push notifications, were described by participants as direct triggers of OCD symptoms.

Rather than removing these features entirely, developers could implement optional design accommodations that give users greater control over their interaction patterns. For example, adjustable interface settings could allow users to disable streak counters, hide quantified feedback (eg, progress bars and daily goals), or consolidate notifications into scheduled digests. Content algorithms might include transparency dashboards where users can view or modify personalization parameters, thereby increasing predictability and reducing uncertainty-driven compulsions. Similarly, introducing a “low-stimulation” or “minimal-feedback” mode could help reduce inadvertent reinforcement of repetitive checking behaviors.

An “OCD-friendly” design, in this context, would prioritize user autonomy, predictability, and control over feedback loops that otherwise promote compulsive engagement. These accommodations could be incorporated without removing core functionality but by expanding the range of interaction options available to all users.

Finally, the mental health technology sector, particularly apps targeting wellness, mindfulness, or productivity, could benefit from evaluating whether their engagement metrics inadvertently reinforce obsessive-compulsive symptom cycles. Collaborating with clinicians, behavioral scientists, and individuals with lived experience of OCD could help establish design standards that promote sustained engagement without exacerbating compulsive behaviors.

### Implications for Researchers

This study opens several avenues for future research. There is a clear need to develop and validate new measurement tools that specifically assess the intersection of OCD symptoms and technology use. These tools could help clinicians differentiate between typical digital habits and pathological compulsions, especially in younger or digitally native populations.

Future studies might also explore how different OCD subtypes respond to specific forms of technology in more controlled, quantitative settings. For example, are individuals with NJR symptoms more sensitive to gamified or goal-based interfaces than those with contamination concerns? Are certain design features (eg, infinite scroll and intermittent rewards) more strongly associated with symptom exacerbation in OCD than in other clinical populations?

Finally, longitudinal studies can address several outstanding questions. First, for individuals with a diagnosis of OCD, what is the time course and progression of technology-related symptoms? Cohort studies of individuals with OCD that include careful assessment for this symptom domain could improve our understanding of the evolution of technology-related obsessions and compulsions. Relatedly, our results demonstrate positive and negative aspects of technology use in adults with OCD; understanding how, and over what period, a neutral or beneficial use of technology transitions into problematic or compulsive use can aid in assessment and evaluation in clinical settings, as well as inform technology design. Finally, tracking technology use in the general population and monitoring for transition to compulsive use could help in identifying specific aspects of the technology and person that may predispose them to these behaviors. Studies in this domain could also assess for conversion to meeting formal OCD diagnostic criteria and evaluate progression of broader OCD symptoms in this population. Overall, understanding these dynamics will be essential as technology continues to evolve and embed itself more deeply into everyday life.

### Limitations

This study has several limitations that should be considered when interpreting its findings. First, the demographics of our participants skewed young and were geographically concentrated in California. The median age of participants was 26.2 (range 20.1‐63.6) years, 14 of the 24 participants resided in California, and 10 participants resided in other states. This geographic concentration likely reflects our recruitment strategies. Seven participants were drawn from other studies conducted at the University of Southern California that required in-person participation in Los Angeles. Additional participants were recruited online through local and national chapters of OCD advocacy organizations. The relatively young age of our sample may, in part, reflect these online recruitment strategies, as previous research has shown that younger adults spend more time online than older adults [26]. Other studies using online recruitment methods have also reported similarly young average participant ages [27-29]. The convenience sampling strategy we used may have contributed to this demographic profile. Regardless of the age profile and geographic distribution of our sample, qualitative research is designed to explore experiences, perspectives, and contexts in depth, rather than to produce findings that are statistically generalizable to a larger population. Its value lies in uncovering rich, detailed insights and generating understanding of complex phenomena, which can then inform theory, practice, or further research.

Second, our participant sample needed a baseline level of technological fluency. All participants were able to access digital recruitment materials, use Zoom for interviews, and speak fluently about their technology use. As a result, the findings may not fully capture the experiences of individuals with lower digital literacy, limited access to technology, or differing generational relationships with digital tools. Individuals for whom technology is so distressing that they cannot consistently use the internet or Zoom would also not be captured in our study.

Additionally, while participants self-identified as having a diagnosis of OCD, we did not conduct formal clinical assessments or structured diagnostic interviews to confirm a diagnosis of OCD or assess specific symptom subtypes. As such, some participant narratives may reflect overlapping symptomatology with related conditions, such as generalized anxiety. However, the use of self-reported diagnosis is an established approach in qualitative mental health research, where the analytic focus is on lived experience, identity, and help-seeking rather than diagnostic validation. Qualitative studies routinely use a self-reported mental health diagnosis as an inclusion criterion for participation, with these diagnoses ranging from OCD to depression to bipolar disorder [30-33]. In line with these precedents, our sampling strategy is appropriate for experiential inquiry, while still requiring cautious interpretation regarding the boundaries between OCD and overlapping or comorbid forms of distress. Additionally, research personnel reviewed transcripts for content consistent with obsessive and compulsive behaviors.

Our analysis was guided by a constructivist grounded theory approach, and results reflect the perspectives and interpretive lens of a research team composed primarily of nonclinicians. While this multidisciplinary team allowed for rich interpretive dialog, the extrapolation of findings was based on analytic interpretation that considered the existing literature on OCD but did not rely on a structured, validated metric. Specifically, aligning participant accounts with disturbing-thought–based or symmetry-based domains was an interpretive process shaped by reflexivity of researchers. Additionally, apparent ambiguity in the data may reflect other barriers to participant disclosure, such as limited insight or the stigmatization of OCD symptoms, rather than the absence of an experience.

Despite these limitations, the study provides novel insights into how individuals with OCD perceive and engage with technology and offers a foundation for future research to more systematically examine these dynamics.

### Conclusions

This study highlights how digital technologies can both trigger and sustain OCD symptom patterns. Participants’ accounts reveal that the design of these systems often mirrors OCD’s own dynamics of uncertainty, reassurance-seeking, and control, blurring the boundary between pathology and platform. Clinically, these findings underscore the importance of assessing technology use as part of symptom formulation and helping patients develop strategies for intentional, rather than compulsive engagement. For designers and digital health developers, we highlight the need for interfaces that minimize reinforcement of compulsive behaviors and allow for user control over triggering features. More broadly, understanding OCD within its technological context reframes it as a disorder increasingly expressed through the architectures of modern attention, emphasizing the shared responsibility of clinicians, researchers, and technologists in shaping environments that support, rather than exploit, cognitive vulnerability.

## Supplementary material

10.2196/85033 Multimedia Appendix 1 Study team demographics.

10.2196/85033 Multimedia Appendix 2 Example semistructured interview guide.

10.2196/85033 Multimedia Appendix 3 Sample coding tree.

10.2196/85033 Multimedia Appendix 4 Table of thematic saturation.

## ABBREVIATIONS

COREQ: Consolidated Criteria for Reporting Qualitative Research
HIPAA: Health Insurance Portability and Accountability Act
IOCDF: International OCD Foundation
NJR: not-just-right
OCD: obsessive-compulsive disorder
REDCap: Research Electronic Data Capture
