# Supplementary material for: Interactions of Technology and Obsessive-Compulsive Disorder Symptomatology in Adults: Qualitative Interview Study
Source: J Med Internet Res. 2026 Feb 5;28:e85033. doi: 10.2196/85033 (PMC12875565; doi:10.2196/85033)
Supplement: Multimedia Appendix 1 [file jmir-v28-e85033-s001.docx]

Appendix 1. Demographic and training characteristics of the research team members and their involvement in conducting qualitative interviews.

| Initials | Education/Occupation | Gender | Conducted Interviews |
| --- | --- | --- | --- |
| ACF | MD, PhD | Man | Yes |
| LO | Medical student | Non-binary | Yes |
| EL | Undergraduate student | Woman | Yes |
| KSP | Undergraduate student | Woman | Yes |
| TV | Medical student | Man | Yes |
| MM | Undergraduate student | Woman | Yes |
| TBAE | Undergraduate student | Man | Yes |
| ES | Undergraduate student | Woman | Yes |
| KP | Medical student | Woman | No |
| HW | Medical student | Man | No |
| DM | Medical student | Man | No |
| UP | Graduate student | Man | No |
| NB | Undergraduate student | Woman | No |
| TBAI | Graduate student | Man | No |
| ZE | Undergraduate student | Woman | No |
| JK | Undergraduate student | Woman | No |
| DS | Medical student | Man | No |
