# Supplementary material for: Interactions of Technology and Obsessive-Compulsive Disorder Symptomatology in Adults: Qualitative Interview Study
Source: J Med Internet Res. 2026 Feb 5;28:e85033. doi: 10.2196/85033 (PMC12875565; doi:10.2196/85033)
Supplement: Multimedia Appendix 2 [file jmir-v28-e85033-s002.docx]

| **SAMPLE INTERVIEW QUESTIONS** |
| --- |
| - Walk me through how you use smartphone apps on a typical day? (IF NO CURRENT/PAST USE, SKIP TO BOTTOM) - Tell me about your experience using apps or smartphone features for HEALTHCARE?   - - Physical health?     - Mental health?     - For OCD?     - How about apps in the past (IF NOT CURRENTLY USING)?   IF HISTORY OF USE (current or past):   - What were your **motivations** to start using? To stop using？ - What **benefits** have you experienced? - What **barriers, difficulties** do you have with using apps? - What **concerns** do you have? - Have your **usage patterns** changed? - What caused these changes? - How did you **learn about** this app?   - Describe any conversations you’ve had about this app. - How has your smartphone and apps **interacted with your OCD**?   - PROBES: change in **symptom level**, change in **symptom frequency**, change is **type of OCD** symptoms   IF NO HISTORY OF USE:   - Are there any **reasons for not trying** them? - "Can you share any insight you have about how other people with OCD are using smartphone apps for their health?" |
| - Walk me through how you use wearable devices on a typical day? (IF NO CURRENT/PAST USE, SKIP TO BOTTOM) - Tell me about your experience using wearable devices for healthcare?   - - Physical health?     - Mental health?     - For OCD?     - How about devices in the past (IF NOT CURRENTLY USING)?   IF HISTORY OF USE (current or past):   - What were your **motivations** to start using? To stop using？ - What **benefits** have you experienced? - What **barriers, difficulties** do you have with using wearables? - What **concerns** do you have? - Have your **usage patterns** changed?   - What caused these changes? - How did you **learn about** this wearable?   - Describe any conversations you’ve had about this wearable. - How has the wearable device **interacted with your OCD**?   - PROBES: change in **symptom level**, change in **symptom frequency**, change is **type of OCD** symptoms   IF NO HISTORY OF USE:   - Are there any reasons for not trying them? - What do you know about how other people with OCD are wearable devices for their health? |
| - Are there other forms of technology that you feel have interacted with your OCD? In what format? (**work communications, social media, internet,** e-system, screens, etc.) - "How would you feel if your doctor or therapist used AI to adjust your medication or guide therapy?" - “What type of engagement in the OCD community has technology allowed you?” - Final thoughts + Questions for us + Additional questions? |
