# Supplementary material for: Interactions of Technology and Obsessive-Compulsive Disorder Symptomatology in Adults: Qualitative Interview Study
Source: J Med Internet Res. 2026 Feb 5;28:e85033. doi: 10.2196/85033 (PMC12875565; doi:10.2196/85033)
Supplement: Multimedia Appendix 3 [file jmir-v28-e85033-s003.docx]

Appendix 3: Sample Coding Tree

Global Codes

**New Finding**

- For data that is important to our research questions, or is interesting, but is not captured by current codes.

**Good Quote**

**Parent Code**

**Child Code**

- **Reasoning one's disengagement with, or abstention from healthcare technology**
  - **Barriers:** Describing difficulties with access or barriers to use of technology as intended.
  - **Functionality:** Describing the intended use of a technology as undesirable, insufficient, etc.
  - **Concerns (other)**: Describing concerns not captured by barriers or functionality (IE data safety, personal image, etc)
- **Exacerbating/enabling OCD symptoms with Technology:** Capturing instances where technology usage intensifies or facilitates OCD symptoms.
  - **Compulsions:** References to repetitive, ritualistic behaviors that are performed using technology. The behavior must be explicitly or contextually described as compulsive or symptomatic by the participant.
  - **Obsessions:** Mentions of intrusive, persistent thoughts or worries exacerbated by technology use. It must be explicitly or contextually described as obsessive or symptomatic by the participant.

- **Alleviating Mental Health/ OCD symptoms with Technology:** Capturing instances where technology, or aspects of a technology help to reduce or alleviate mental health or OCD symptoms.
- **Exploring the role(s) of technology and Mental Healthcare**: Capturing discussions about how technology is used within the context of OCD healthcare, both practically and theoretically.
  - **Theoretical desires for technology in Mental/ OCD Healthcare:** Discussions on the potential uses or conceptual roles technology could play in OCD care.
  - **Experiences with technology in Mental/OCD Healthcare:** References to how technology has been integrated or experienced in participant’s OCD healthcare journey. This can encompass learning/understanding of OCD, diagnosis, monitoring, treatment/therapy, etc.
- **Engaging or abstaining in community through technology**: Descriptions of how individuals with OCD either use technology to connect with others in communities that focus on mental health or OCD or describe their hesitancies towards these connections. Can encompass joining online support groups, forums, social media, etc.
