# Supplementary material for: Interactions of Technology and Obsessive-Compulsive Disorder Symptomatology in Adults: Qualitative Interview Study
Source: J Med Internet Res. 2026 Feb 5;28:e85033. doi: 10.2196/85033 (PMC12875565; doi:10.2196/85033)
Supplement: Multimedia Appendix 4 [file jmir-v28-e85033-s004.docx]

Appendix 4: Saturation Tracking Using the Global “New Finding” Code

| Interview # | "New finding" applications |
| --- | --- |
| 1-5 | N/A |
| 6 | 0 |
| 7 | 0 |
| 8 | 2 |
| 9 | 1 |
| 10 | 0 |
| 11 | 1 |
| 12 | 3 |
| 13 | 0 |
| 14 | 0 |
| **15** | **1** |
| 16 | 0 |
| 17 | 0 |
| 18 | 0 |
| 19 | 0 |
| 20 | 0 |
| 21 | 0 |
| 22 | 0 |
| 23 | 0 |
| 24 | 0 |

Appendix 4 summarizes use of the global “New Finding” code, applied when data relevant to the research questions were not captured by existing codes. The absence of new applications after interview 15 indicates that additional data were increasingly redundant with previously coded material, marking the point of inductive thematic saturation. Interviews 1–5 were conducted during the open-coding phase, when all relevant data were captured through initial code generation rather than through the global “New Finding” code.
